# Structure-Antioxidative and Anti-Inflammatory Activity Relationships of Purpurin and Related Anthraquinones in Chemical and Cell Assays

**DOI:** 10.3390/molecules22020265

**Published:** 2017-02-10

**Authors:** Woo Nam, Sung Phil Kim, Seok Hyun Nam, Mendel Friedman

**Affiliations:** 1 Department of Biological Science, Ajou University, Suwon 16499, Korea; storyhunter@ajou.ac.kr; 2 Research Institute of Basic Sciences, Ajou University, Suwon 16499, Korea; sp-1108@hanmail.net; 3 STR Biotech. Ltd., Chuncheon 24232, Korea; 4 Western Regional Research Center, Agricultural Research Service, U.S. Department of Agriculture, Albany, CA 94710, USA

**Keywords:** purpurin, anthraquinones, BHA, antioxidant tests, macrophage cells, reactive oxygen species, inflammasome activation, anti-inflammatory, cytokines, health benefits

## Abstract

Anthraquinone (9,10-anthraquinone) and several hydroxy derivatives, including purpurin (1,2,4-trihydroxyanthraquinone), anthrarufin (1,5-dihydroxyanthraquinone), and chrysazin (1,8-dihydroxyanthraquinone), were evaluated for antioxidative and anti-inflammatory activities in chemical assays and mammalian cells (murine macrophage RAW 264.7 cells). Several tests were used to assess their activities: 1,1-diphenyl-2-picrylhydrazyl (DPPH) free radical; ABTS radical cation; hydrogen peroxide scavenging; reduction of potassium ferricyanide; chelation of ferrous ions; inhibition of lipid peroxidation; inhibition of nitric oxide generation; scavenging of the intracellular hydroxyl radical; expression of NLRP3 polypeptide for inflammasome assembly; and quantitation of proinflammatory cytokine interleukin 1β (IL-1β) for inflammasome activation. The results show that purpurin, from the root of the madder plant (*Rubia tinctorum* L.), exhibited the highest antioxidative activity in both chemical and cultured cell antioxidant assays. The antioxidative activities of the other three anthraquinones were lower than that of purpurin. In addition, purpurin could down-regulate NLRP3 inflammasome assembly and activation, suggesting that it might protect foods against oxidative damage and prevent in vivo oxidative stress and inflammation. Structure-activity relationships and the significance of the results for food quality and human health are discussed.

## 1. Introduction

Anthraquinones and derivatives are present in various plant roots. For example, a review by Xu et al. [1], which includes a discussion of structure-activity relationships, lists 142 quinone derivatives in 12 *Rubia* species. Zengin et al. [2] showed that Asphodeline roots might provide a new source of bioactive quinones and other natural compounds, and Usai and Marchetti [3] describe the distribution of anthraquinones in the *Rubia peregrina* L. plant.

The anthraquinone purpurin is a component of the ancient pigment known as Madder Lake [4]. It occurs as a colorless glycoside in the root of the common madder plant (*Rubia tinctorum* L.) [5,6]. The cellular and molecular basis for the reported health-promoting properties of purpurin in vitro and in vivo seems to be associated with antioxidative [7] and antigenotoxic [8,9] effects that include (a) chemoprotective effects against DNA adduct formation in mice; and (b) inhibition of biological alkylations of DNA by carcinogenic heterocyclic amines, present in heat-processed (cooked) meat and poultry products [10,11,12]. In the present study, we observed that anti-inflammatory and immunomodulating properties in cells might also contribute to the mentioned beneficial effects. In related studies (a) Malterud et al. [13] found that in vitro radical scavenging activities of anthraquinones correlated with the antioxidant activities in hepatocytes, whereas the inhibition of enzymatic lipid peroxidation showed no correlations; (b) Yen et al. [14] reported that (based on observed inhibition of oxidation of linoleic acid emulsion by five anthraquinones, not including purpurin) the relative antioxidative properties seem to depend on both hydroxyl substitution in the structure and on the method of analysis; (c) Baghiani et al. [15] found that purpurin had a radical scavenging effect in the 1,1-diphenyl-2-picrylhydrazyl (DPPH) assay with an IC_50_ = 3.491 μg/mL; (d) Kumar et al. [16] observed using an in vitro assay that alizarin was more effective than purpurin in protecting DNA against damage by hydroxyl radicals; (e) Marković et al. [17] used theoretical modeling experiments to determine the influence of structures of three anthraquinones, not including purpurin, on the mechanism of antioxidative activities involving free radicals; and (f) Özbakır Işın [18] found that the experimental antioxidative properties of four hydroxyanthraquinones paralleled theoretical values calculated using quantum chemical methods.

**Figure 1:**
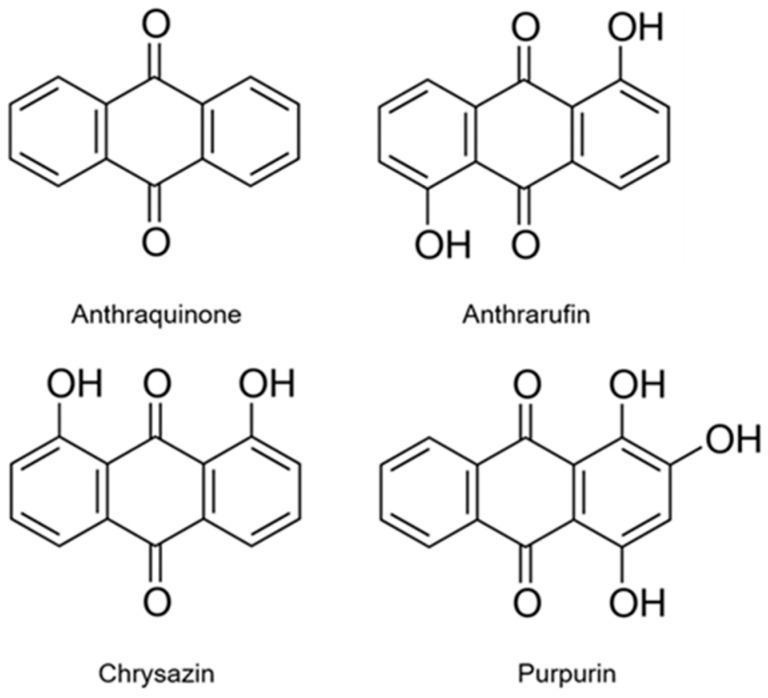
Structure of anthraquinones evaluated in the present study.

The main objective of the present study was to further explore the potential of purpurin to improve food quality and health in comparison with three structurally-related anthraquinones (anthraquinone, anthrarufin, and chrysazin) (Figure 1) and the widely used food antioxidant 3-*tert*-butyl-4-hydroxyanisole (BHA) [19]. To achieve this objective, we used the free-radical-scavenging activity, inhibition of linoleic peroxidation, 2,2’-azinobis-3-ethylbenzothiazoline-6-sulphonic acid (ABTS) radical cation decolorization, hydrogen peroxide scavenging activity, ferricyanide test reducing power, and ferrous ion binding chemical assays, as well the in vitro cell viability 3-(4,5-dimethylthiazol-2-yl)-2,5-diphenyl tetrazolium bromide (MTT) assay, nitric oxide (NO) generation, cell inflammasome induction, quantitation of proinflammatory cytokine, and intracellular hydroxyl radical scavenging cell assays.

## 2. Results and Discussion

### 2.1. Antioxidative Chemical Assays

#### 2.1.1. DPPH Radical Scavenging Activity

The 1,1-diphenyl-2-picrylhydrazyl (DPPH) radical scavenging activity of the four anthraquinone compounds was determined at five concentrations ranging from 1 μM to 250 μM (Table 1). The data show significant decreases in the concentration of DPPH radical due to the scavenging by the test compounds. Although all test compounds were active in the assay, purpurin showed the highest value for DPPH radical scavenging. This is confirmed by their IC_50_ values (the lower the value the greater the activity) that show that the activity decreased in the order of purpurin > chrysazin > anthraquinone > anthrarufin, although only purpurin was statistically different.

#### 2.1.2. Inhibition of Lipid Peroxidation

Since lipid peroxidation is known to damage food ingredients, such as fats, and is one of the major oxidative stresses in cell systems in vivo, the inhibitory effect of the compounds on lipid peroxidation was assessed using a linoleic acid peroxidation assay. The results presented in Table 2 show that of the four anthraquinones, purpurin had the highest inhibitory activity against lipid peroxidation; an activity that was similar to that of BHA.

#### 2.1.3. ABTS Radical Cation Scavenging Activity

Total antioxidant activity of the compounds was measured again using the ABTS radical cation decolorization assay, known as an excellent tool for determining the antioxidant activity of hydrogen-donating and of chain-breaking antioxidants. Table 3 shows that the IC_50_ of ABTS radical cation scavenging activity of purpurin could not be compared to the other three anthraquinones because we were not able to determine their IC_50_ values. The calculated IC_50_ also show that purpurin’s potency in ABTS radical cation scavenging is about 3.2-fold lower that observed with BHA.

#### 2.1.4. Hydrogen Peroxide Scavenging Activity

Table 4 shows that purpurin exhibited the highest hydrogen peroxide scavenging activity. The reduction in peroxide by purpurin was similar that of BHA. Anthrarufin, chrysazin, and anthraquinone were inactive in this assay at all five concentrations. Although we failed to determine IC_50_ values for chrysazin and anthraquinone, Table 4 shows that purpurin is a potent hydrogen peroxide scavenging agent with an activity that is about the same as that observed with BHA.

#### 2.1.5. Reducing Power Measured by Ferricyanide Assay

The antioxidant activity of the four compounds was evaluated by measuring the abilities of the test compounds to reduce potassium ferricyanide (Fe^3+^) to the ferrocyanide (Fe^2+^) state [20] Table 5 shows data for the extent of reduction in terms of absorbance values at 700 nm for five concentrations of the five test compounds including the positive control BHA. The results show that (a) the reducing power of purpurin was higher than those of anthrarufin, chrysazin, and anthraquinone (1.45 vs. 0.06, 0.02 and 0.03 at 250 μM, respectively); and (b) that its activity of purpurin is about the same as that of control antioxidant BHA.

We also examined the extent of chelation of ferrous sulfate by the test compounds because chelation of ferrous ions can inhibit hydroxyl radical formation [21,22]. EDTA, a well-known divalent cation chelating agent, was used as control. The results show that because at 250 μM the extent of complex formation was extremely low among anthraquinones compared to that of EDTA (Table 6), it was not possible to determine an IC_50_ value. However, the data suggest that the chelating ability of purpurin on ferrous ions does not seem to influence its action of reducing the ferricyanide to the ferrocyanide oxidation state, suggesting that purpurin can both inactivate free radicals as well as prevent formation of radicals by ferrous ions.

### 2.2. Antioxidative and Anti-Inflammatory Cell Assays

#### 2.2.1. Effects on NO Production and Cell Viability in Murine RAW 264.7 Macrophage Cells

**Figure 2:**
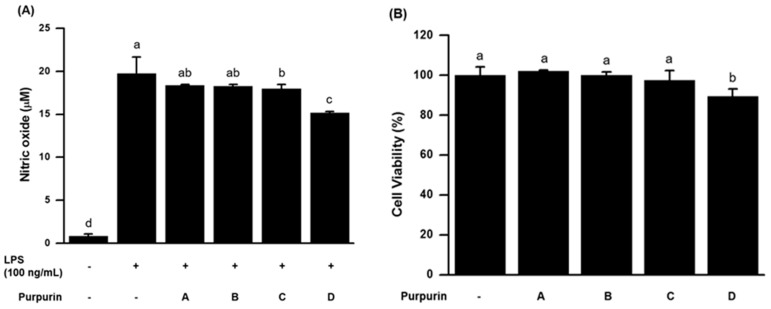
Effect of purpurin on NO production in RAW 264.7 murine macrophage cells. (**A**) The cells stimulated with LPS were treated with various concentrations of purpurin: A, 1 μM; B, 10 μM; C, 25 μM; D, 50 μM. LPS alone was used as a control; (**B**) Cell viability changes. The cells were treated with various concentrations of purpurin for 48 h and then cell viability was measured by MTT assay. Data are expressed as the mean ± SD (*n* = 3). Bars not sharing a common letter are significantly different between groups at *p* < 0.05.

Since nitric oxide (NO), mainly produced from activated macrophages, is involved in innate immunity as a toxic agent against pathogenic bacteria and viruses, through the action of peroxinitrite or modulation of cell physiology [23,24,25], we examined the modulatory effect of purpurin on NO production in RAW 264.7 murine macrophage cells. As depicted in Figure 2A, lipopolysaccharide (LPS)-stimulated NO production was significantly suppressed by 50 μM purpurin treatment (~23% suppression compared with LPS-treated control). To rule out the possibility that the suppressive effect was caused by purpurin-induced cell death, cell viabilities at indicated dosed of purpurin were determined by the widely used MTT cytotoxicity assay. Figure 2B shows that cell viability was largely unaffected by purpurin at concentrations <50 μM. Even at 50 μM, the viability decreased only by ~11%, indicating that purpurin, itself, was not cytotoxic in the macrophage cells.

#### 2.2.2. Suppression of Cellular Oxidative Stress

**Figure 3:**
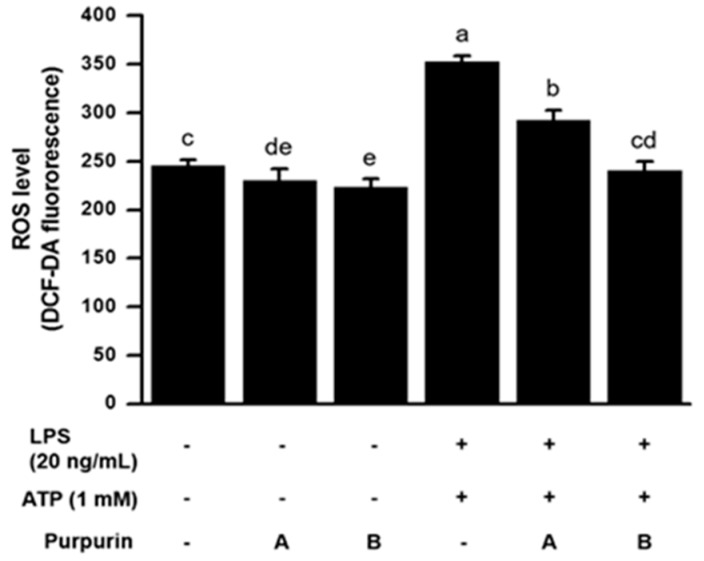
Effect of purpurin on intracellular ROS levels in activated RAW 264.7 murine macrophage cells. The cells, consecutively stimulated with LPS for 3 h and then ATP for 30 min, were treated with DCF-DA (20 μM) and various concentrations of purpurin for 30 min: A, 10 μM; B, 50 μM. The resultant fluorescent DCF levels were measured. Data are expressed as the mean ± SD (*n* = 3). Bars not sharing a common letter are significantly different between groups at *p* < 0.05.

The results obtained from the in vitro chemical assays clearly demonstrated that purpurin was the strongest antioxidant among the compounds examined. It was, therefore, of interest to determine whether the strong antioxidant activity would also operate in a cell milieu. Figure 3 shows the scavenging ability of purpurin on intracellularly generated-hydroxyl radicals in activated RAW 264.7 cells. The data indicate that purpurin significantly and effectively decreased the intracellular hydroxyl radical level in a dose-dependent manner (~33% scavenging at 50 μM compared with the LPS plus adenosine 5′-triphosphate (ATP)-activated control). This result demonstrates the potency of purpurin to lower intracellular oxidative stress through scavenging of reactive oxygen radicals (ROS).

#### 2.2.3. Inhibition of Inflammasome Formation

**Figure 4:**
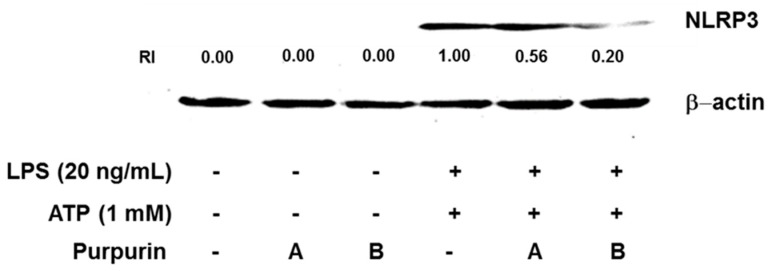
Western blot analysis for determination of regulatory effect of purpurin on NLRP3 expression in activated RAW 264.7 murine macrophage cells. The cells, consecutively stimulated with LPS for 3 h and then ATP for 30 min, were treated with various concentrations of purpurin for 30 min: A, 10 μM; B, 50 μM. Total cell lysates were prepared and subjected to Western blot. RI (relative intensity) was defined as the ratio of band intensity of target protein to those of β-actin. The blot pattern is representative of three independent experiments.

**Figure 5:**
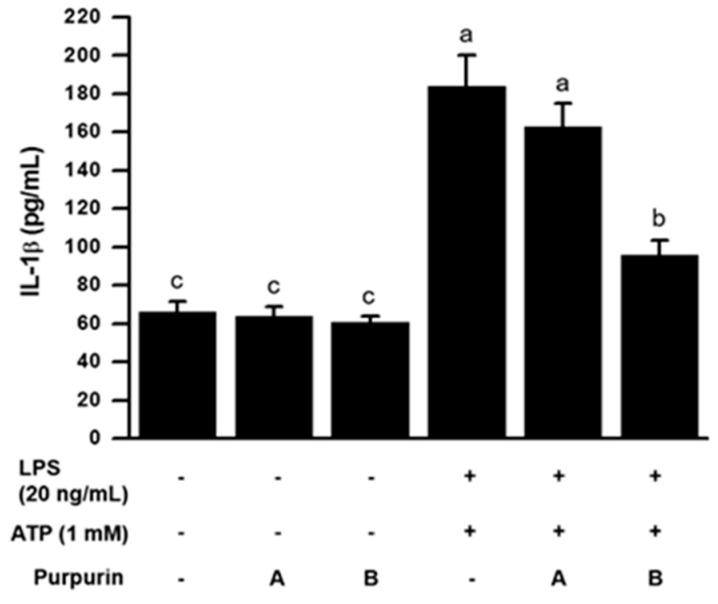
Effect of purpurin on IL-1β production in activated RAW 264.7 murine macrophage cells. The cells, consecutively stimulated with LPS for 3 h and then ATP for 30 min, were treated with various concentrations of purpurin for 30 min: A, 10 μM; B, 50 μM. Then the culture media were recovered and IL-1β secretion was measured by ELISA assay. Data are expressed as the mean ± SD (*n* = 3). Bars not sharing a common letter are significantly different between groups at *p* < 0.05.

Inflammasomes have been reported to be involved in both inflammation and programmed cell death [26,27,28]. Because intracellular ROS generation has been explored as one of the factors for NLRP3 inflammasome activation, we first examined, using Western blot analysis, whether purpurin treatment will lead to down-regulation of the inflammasome assembly scaffold and activation in RAW 264.7 cells [29]. The data in Figure 4 indicate that NLRP3 expression was clearly decreased by the purpurin treatment in a dose dependent manner (~44% and 80% decreases at 10 μM and 50 μM, respectively). We also investigated, using an enzyme-linked immunosorbent assay (ELISA), the caspase-1-triggered maturation and secretion of interleukin (IL-1β) by quantitation of the cytokine in the cell culture supernatant. The data in Figure 5 also show decreases in IL-1β secretion upon purpurin treatment in a dose-dependent manner (~11% and 48% decreases at 10 μM and 50 μM, respectively, compared with the LPS plus ATP-activated control). These observations demonstrate the anti-inflammatory ability of purpurin to suppress assembly and activation of the NLRP3 inflammasome through lowering of intracellular antioxidative stress.

#### 2.2.4. Structure-Activity Aspects

As noted elsewhere for phenolic compounds [30], the mechanism of the antioxidative effect involves abstraction by the antioxidant of a high- energy reactive free electron from free radicals. The electron is then dissipated (delocalized) within the phenolic OH groups and the aromatic ring of the antioxidant to a less reactive (lower energy; less damaging) free radical.

Figure 1 shows that the four evaluated anthraquinones differ only in the number of OH groups attached to the aromatic anthraquinone moiety. These range from zero for anthraquinone, to two for anthrarufin and chrysazin, and three for purpurin. Moreover, the OH groups are located at different positions on the three molecules. The cited experimental data suggest that the number and location of the OH groups seems to be largely responsible for the observed large variations in the trends of antioxidative properties. Purpurin with three OH groups, two of which are located in the ortho position of the anthraquinone molecule, exhibited the highest activity in all assays. Since the anthraquinone molecule without OH groups was also active in the chemical assays, it seems the quinone oxygens of the central rings of the molecules also participate in the mechanism of the described antioxidative and properties.

The cited observations strikingly demonstrate the strong antioxidative potential of purpurin as determined using five independent antioxidant chemical antioxidant assays. The results of the cell assays also presented here reinforce this potential in cellular environments and also demonstrate for the first-time strong anti-inflammatory properties of purpurin. Purpurin was not toxic to the cells. The antioxidative properties of purpurin are a result of its ability to destroy free radicals; such free radicals can damage or spoil fat-containing food, for example, soybean oil and meat. Purpurin could, therefore, have potential as a food additive to provide both antioxidative and anti-inflammatory properties. Indeed, cell data suggest that purpurin also has the potential to destroy reactive oxygen species that damage DNA and essential proteins and to suppress inflammation via down-regulation of inflammasome assembly and activation, suggesting its further potential value to protect against multiple chronic and metabolic diseases associated with inflammation. The potential of purpurin to improve food quality and safety and human health merits further study, provided that it can be shown that is safe for consumption. The recently validated pharmacokinetic method showed that a purpurin-containing plant extract administered orally to rats is slowly absorbed and metabolized in the plasma, suggesting that this might be a useful approach (model) for further studies on the pharmacology and toxicology of purpurin and related anthraquinones [31].

## 3. Materials and Methods

### 3.1. Materials

Anthraquinone (9,10-anthraquinone), anthrarufin (1,5-dihydroxyanthraquinone), chrysazin (1,8-dihydroxy-9,10-anthraquinone), purpurin (1,2,4-trihydroxyanthraquinone), 1,1-diphenyl-2-picrylhydrazyl (DPPH), 2,2′-azinobis-3-ethylbenzothiazoline-6-sulphonic acid (ABTS), 3-*tert*-butyl-4-hydroxyanisole (BHA), 3-(4,5-dimethylthiazol-2-yl)-2,5-diphenyl tetrazolium bromide (MTT), lipopolysaccharide (LPS, *Escherichia coli* 0111:B4), and other analytical grade reagents were obtained from Sigma-Aldrich (St. Louis, MO, USA). 2′,7′-Dichlorofluorescein diacetate (DCF-DA) was purchased from Calbiochem (San Diego, CA, USA). Dulbecco’s modified Eagle’s medium, phosphate-buffered saline, fetal bovine serum, and other miscellaneous cell culture reagents were purchased from Hyclone Laboratories (Logan, UT, USA).

### 3.2. Antioxidative Chemical Tests

#### 3.2.1. DPPH Free Radical Scavenging Assay

The antioxidant activities of the four anthraquinones and of BHA were measured in terms of hydrogen donating or radical scavenging ability according to the method of Mathew and Abraham [32]. The samples in DMSO were appropriately diluted with ethanol, and the sample (0.5 mL) was mixed with 100 mM sodium acetate (0.25 mL, pH 5.5). An ethanol solution (0.25 mL) of DPPH (200 μg/mL) was then added to the mixture. After 30 min at room temperature, the absorbance of the reaction mixtures was read at 517 nm using a UV-VIS spectrophotometer (model T60, PG Instruments, Leicester, UK). Commercial antioxidant BHA was employed as a positive control. The scavenging of DPPH radical by the compounds was calculated according to the following formula:

DPPH scavenging activity (%) [((*Ac*_0_ − *Ac*_1_) − (*As*_0_ − *As*_1_))/(*Ac*_0_ − *Ac*_1_)] × 100 (1)

where *Ac*_0_ equals the absorbance of the control with DPPH only; *Ac*_1_, the absorbance of the control without DPPH; *As*_0_, the absorbance in the presence of sample with DPPH; and *As*_1_,: the absorbance in the presence of sample without DPPH.

#### 3.2.2. Inhibition of Linoleic Acid Peroxidation

The in vitro antioxidant activity of the four compounds was determined by the thiocyanate method described by Mitsuda et al. [33]. Briefly, linoleic acid emulsion was prepared to contain 0.00028% linoleic acid and 0.00035% Tween-20 in 0.02 M phosphate buffer (pH 7.0). The samples dissolved in DMSO at various concentrations were mixed with 100 volumes of the linoleic acid emulsion. The mixture was then placed in the dark at 37 °C for 30 h to accelerate lipid peroxidation. Ferrous chloride (0.02 M in 3.5% HCl) and ammonium thiocyanate (30% *w*/*v*) were then added and the peroxidation value was measured by reading the absorbance of the resulting chromogen at 500 nm. The inhibition of lipid peroxidation was calculated according to the following formula:

Inhibition (%) = [((*Ac*_0_ − *Ac*_1_) − (*As*_0_ − *As*_1_))/(*Ac*_0_ − *Ac*_1_)] × 100 (2)

where *Ac*_0_ equals the absorbance of the control; *Ac*_1_, the absorbance of the control without ferrous chloride and ammonium thiocyanate; *As*_0_, absorbance in the presence of sample; and *As*_1_, the absorbance in the presence of sample without ferrous chloride and ammonium thiocyanate.

#### 3.2.3. ABTS Radical Cation Decolorization Assay

The ABTS decolonization assay, which involves the generation of the ASBTS+ chromophore by the oxidation of ABTS with potassium persulfate that is applicable for both hydrophilic and hydrophobic compounds, was carried out according to the method described by Re et al. [34]. Briefly, 7 mM ABTS solution in 2.45 mM potassium persulfate was diluted with ethanol to an absorbance of 0.7 ± 0.002 at 734 nm. Each sample was then added to the diluted ABTS solution (1 mL). After 30 min incubation at room temperature, the absorbance of the compounds was read at 734 nm. The scavenging of ABTS radical cation by the compounds was calculated according to the following formula:

ABTS radical scavenging activity (%) = [((*Ac*_0_ − *Ac*_1_) − (*As*_0_ − *As*_1_))/(*Ac*_0_ − *Ac*_1_)] × 100 (3)

where *Ac*_0_ equals the absorbance of the control with ABTS only; *Ac*_1_, the absorbance of control without ABTS; *As*_0_, the absorbance in the presence of sample with ABTS; and *As*_1_, the absorbance in the presence of sample without ABTS.

#### 3.2.4. Hydrogen Peroxide Scavenging Assay

Hydrogen peroxide (H_2_O_2_) scavenging activity was determined according to the literature methods [35,36]. The four samples dissolved in phosphate-buffered saline (pH 7.0) (100 μL) at various concentrations were added to the same volume of 0.002% H_2_O_2_. Phosphate buffer and 100 mM NaCl (0.8 mL of 0.1 M) were then added to this solution. The reaction mixture was preincubated for 10 min at 37 °C. The phenol red dye (1 mL; 0.2 mg/mL) with horseradish peroxidase (0.1 mg/mL; 0.1 M) in phosphate buffer was the added. After 15 min incubation, 1 M NaOH (100 μL) was added and the absorbance was measured at 610 nm immediately. The hydrogen peroxide scavenging activity was calculated according to the following formula:

Hydrogen peroxide scavenging (%) = [((*Ac*_0_ − *Ac*_1_) − (*As*_0_ − *As*_1_))/(*Ac*_0_ − *Ac*_1_)] × 100 (4)

where *Ac*_0_ equals the absorbance of the control; *Ac*_1_, the absorbance of the control without phenol red; *As*_0_, absorbance in the presence of sample; and *As*_1_, the absorbance in the presence of sample without phenol red.

#### 3.2.5. Ferricyanide Reducing Power Assay

The reducing power of the samples was determined according to the method of Oyaizu [20], with some modifications. In brief, each compound solution at various concentration was diluted with 0.7 mL of 50 mM phosphate buffer (0.7 mL, 50 mM, pH 7.0). The diluted sample was then mixed with 0.5 mL of 1% potassium ferricyanide [K_3_Fe(CN)_6_] and the mixture was incubated at 50 °C for 20 min. To this mixture was then added trichloroacetic acid (0.5 mL, 10%) followed by centrifugation at 3000 rpm for 10 min. Aliquot (0.5 mL) from the upper layer of the solution was then mixed with the same volume of deionized water plus 0.1% FeCl_3_, and the absorbance measured at 700 nm.

#### 3.2.6. Ferrous Ion Binding Assay

The chelation of ferrous ions by the samples was estimated by the method reported by Dinis et al. [37]. The samples were added to FeCl_2_ (1 mL, 0.05 mL). The reaction was initiated by the addition of ferrozine (1 mM, 0.1 mL). After thorough mixing, the reaction mixture was left standing at room temperature for 10 min. After the reaction mixture had reached equilibrium, the absorbance was then read spectrophotometrically at 562 nm. The inhibition of ferrozine-Fe^2+^ complex formation was calculated according to the following formula:

Inhibition (%) = [((*Ac*_0_ − *Ac*_1_) − (*As*_0_ − *As*_1_))/(*Ac*_0_ − *Ac*_1_)] × 100 (5)

where *Ac*_0_ equals the absorbance of the control; *Ac*_1_, the absorbance of the control without ferrozine; *As*_0_, absorbance in the presence of sample; and *As*_1_, the absorbance in the presence of sample without ferrozine.

### 3.3. Cell Assays for Antioxidative and Anti-Inflammatory Activities

#### 3.3.1. Cell Culture

The RAW264.7 murine macrophage cell line from the American Type Culture Collection (ATCC; Manassas, VA, USA) was cultured in Dulbecco’s modified Eagle’s medium supplemented with 10% heat-inactivated fetal bovine serum containing 100 U/mL penicillin and 100 μg/mL streptomycin. Cells were cultured at 37 °C in a humidified atmosphere with 5% CO_2_. The medium was replaced every three days until the cell reached 70%~80% cell density.

#### 3.3.2. Cell Viability MTT Assay

MTT staining was employed to assess the cytotoxic effects of purpurin [38]. Briefly, the RAW 264.7 cells were seeded into 96-well plate at a density of 1 × 10^4^ cells/well, and then the cells were treated with purpurin at the indicated concentration for 48 h at 37 °C humidified air containing 5% CO_2_. After treatment, the cells were stained by adding MTT. The resultant intracellular chromogen formazan products were solubilized with DMSO. Absorbance of the chromogen was read in a microplate reader (iMark, Bio-Rad, Hercules, CA, USA) at 570 nm at a reference wavelength of 655 nm.

#### 3.3.3. Nitric Oxide Generation Assay

Nitric oxide (NO) was measured by determining the concentration of its stable oxidative metabolite nitrite using a microplate assay as described by Murakami et al. [39]. Briefly, 100 ng/mL LPS (Sigma-Aldrich)-stimulated RAW 264.7 cells (1 × 10^5^ cells/well) in 96-well plate were treated with purpurin at various concentration for 48 h. After incubation, the culture medium was mixed with an equal volume of Griess reagent (1% sulfanilamide and 0.1% *N*-(napthyl)ethylenediamine dihydrochloride in 5% phosphoric acid) at room temperature for 15 min. The absorbance was then measured at 570 nm using a microplate reader (Bio-Rad). Sodium nitrite was used as the standard.

#### 3.3.4. Cell Treatment for Inflammasome Induction

To induce inflammasomes in RAW264.7 cells, the cells were allowed to adhere overnight at a cell density of 1 × 10^5^ cells/well in a 96-well plate and washed with phosphate-buffered saline to remove unattached cells, and then stimulated with LPS (20 ng/mL) for 3 h and 1 mM ATP for additional 30 min. RAW 264.7 cells not treated with LPS and ATP were used as the controls.

#### 3.3.5. Quantitation of Cytokine

The IL-1β cytokine level in supernatant from LPS plus ATP-stimulated RAW 264.7 cell culture was determined using an ELISA kit (Life Technologies, Carlsbad, CA, USA) according to the manufacturer’s instructions.

#### 3.3.6. Intracellular Hydroxyl Radical Scavenging Assay

The scavenging of reactive oxygen species (ROS) by the samples in a cell milieu was performed according to the method of Lin et al. [40], with some modification. RAW 264.7 cells (1 × 10^5^ cells/well) in a 96-well plate were stimulated successively with 20 ng/mL LPS and 1 mM ATP as described above, followed by the treatment with a nonfluorescence probe DCF-DA (20 μM) at the indicated concentration of the samples at 37 °C for 30 min. The cells were then washed trice with phosphate-buffered saline, and the highly fluorescent DCF level, produced from DCF-DA through oxidation by hydroxyl radical within the cell, was measured immediately using a fluorometer (Spectramax Gemini XS, Molecular Device, Sunnyvale, CA, USA) at 485 and 530 nm excitation and emission wavelengths, respectively.

#### 3.3.7. Western Blot Analysis of Cell Proteins

RAW 264.7 cells were extracted with RIPA (radioimmunoprecipitation assay) buffer (50 mM Tris Cl, 150 mM NaCl, 1% NP-40, 0.5% sodium deoxycholate, 0.1% SDS and 1 mM EDTA, pH 7.4) for preparation of whole cell proteins. Protein concentrations were determined according to the Bradford method using a Bio-Rad Protein kit. Bovine serum albumin was used as standard. The cell extract containing proteins (30 μg) were separated on 10% SDS-polyacrylamide gels and electrophoretically transferred onto a nitrocellulose membrane (Millipore, Billerica, MA, USA). The membrane was blocked in 5% skim milk at 4 °C overnight and probed with the primary antibodies as follows anti-mouse NLRP3 monoclonal antibody (Cell Signaling Tech., Danvers, MA, USA) and anti-mouse β-actin monoclonal antibody (Merck Millipore, Darmstadt, Germany). After the primary antibody reaction for at least 3 h, the secondary antibody reactions with horse radish peroxidase-conjugated anti-rabbit IgG and anti-mouse antibodies, respectively, were performed under the same conditions. Blots were developed using the ECL detection kit (Pierce, Rockford, IL, USA). The intensity of separated protein bands was quantified using a gel documentation system (Model LAS-1000CH, Fuji Photo Film Co., Tokyo, Japan). At least three separate replicates were determined for each experiment.

### 3.4. Statistical Analysis

Results are expressed as the mean ± SD of three independent experiments. Significant differences between means were determined by the ANOVA test using the Statistical Analysis Software package SAS (Cary, NC, USA). *p* < 0.05 is regarded as significant.

## 4. Conclusions

Beneficial effects of antioxidants in foods are due to their ability to destroy reactive, food-damaging free radicals, thus enhancing nutritional quality and safety [41,42]. Beneficial effects of antioxidants in vivo are due to their ability to destroy reactive oxygen species (ROS) that damage DNA and essential enzymes [43]. Since inflammation is a risk factor for aging [44], arthritis [45], asthma in mice [46] and in children [47], cancer [48], as well as diabetes and obesity [49], the results of the present study, and the cited findings by other investigators, suggest that purpurin has the potential to protect food against oxidative damage and ameliorate chronic diseases in vivo, analogous to the protective effects of mushroom polysaccharides described elsewhere [50]. These aspects merit further study.

## Figures and Tables

**Table 1:** DPPH radical scavenging capacities of BHA and anthraquinones *^a^*.

Sample	DPPH Scavenging Activity (%)					
	1 µM	10 µM	50 µM	100 µM	250 µM	IC_50_ (µM)
BHA	1.2 ± 1.9 ^c^	16.4 ± 2.8 ^c^	59.93 ± 0.55 ^b^	65.4 ± 0.40 ^b^	62.1 ± 3.2 ^b^	36.5 ± 1.7 ^b^
Purpurin	8.7 ± 1.3 ^b^	36.8 ± 1.2 ^a^	78.7 ± 3.1 ^a^	88.0 ± 1.9 ^a^	92.5 ± 1.3 ^a^	20.9 ± 1.4 ^b^
Anthrarufin	8.56 ± 0.67 ^b^	18.62 ± 0.26 ^c^	27.5 ± 1.4 ^c^	35.9 ± 2.3 ^c^	58.2 ± 1.1 ^b^	225 ± 19 ^a^
Chrysazin	6.9 ± 2.3 ^b^	9.1 ± 4.0 ^d^	12.0 ± 3.1 ^d^	26.0 ± 7.0 ^d^	59.0 ± 1.7 ^b^	215 ± 14 ^a^
Anthraquinone	21.0 ± 1.2 ^a^	29.0 ± 2.1 ^b^	30.2 ± 1.3 ^c^	37.33 ± 0.31 ^c^	54.2 ± 2.4 ^c^	213 ± 22 ^a^

*^a^* Data are expressed as the mean ± SD (*n* = 3). Values in each column with the same letter are not significantly different between groups at *p* < 0.05.

**Table 2:** Inhibition of linoleic acid peroxidation by BHA and anthraquinones *^a^*.

Sample	Inhibition of Lipid Peroxidation (%)					
	1 µM	10 µM	50 µM	100 µM	250 µM	IC_50_ (µM)
BHA *^b^*	92.2 ± 2.0 ^a^	95.1 ± 1.4 ^a^	98.4 ± 2.5 ^a^	98.9 ± 3.1 ^a^	98.28 ± 0.64 ^a^	0.99 ± 0.01 ^c^
Purpurin	42.9 ± 1.9 ^b^	83.2 ± 1.1 ^b^	96.0 ± 1.1 ^a^	98.35 ± 0.65 ^a^	97.5 ± 1.4 ^a^	1.27 ± 0.05 ^c^
Anthrarufin	22.12 ± 0.83 ^d^	34.7 ± 1.5 ^c^	71.1 ± 2.0 ^b^	85.01 ± 0.59 ^b^	88.11 ± 0.82 ^b^	23.5 ± 1.7 ^c^
Chrysazin	11.9 ± 4.1 ^e^	17.0 ± 1.7 ^d^	41.1 ± 1.4 ^c^	41.54 ± 0.49 ^d^	50.57 ± 0.29 ^d^	202 ± 25 ^a^
Anthraquinone	27.0 ± 1.8 ^c^	33.85 ± 0.55 ^c^	42.5 ± 3.7 ^c^	49.7 ± 2.7 ^c^	54.70 ± 0.21 ^c^	101 ± 21 ^b^

*^a^* Data are expressed as the mean ± SD (*n* = 3); *^b^* IC_50_ value was calculated using additional three inhibition values at 1, 10, and 100 nM BHA. Values in each column with the same letter are not significantly different between groups at *p* < 0.05.

**Table 3:** ABTS radical cation scavenging capacities of BHA and anthraquinones *^a^*.

Sample	ABTS Scavenging Activity (%)					
	1 µM	10 µM	50 µM	100 µM	250 µM	IC_50_ (µM)
BHA	3.03 ± 0.70 ^a^	31.49 ± 0.69 ^a^	98.98 ± 0.04 ^a^	99.22 ± 0.03 ^a^	99.07 ± 0.22 ^a^	17.55 ± 0.13 ^b^
Purpurin	2.49 ± 0.99 ^a^	21.09 ± 0.27 ^b^	50.32 ± 0.41 ^b^	61.21 ± 0.10 ^b^	76.04 ± 0.30 ^b^	54.5 ± 0.6 ^a^
Anthrarufin	1.04 ± 0.68 ^ab^	0.55 ± 0.45 ^c^	1.4 ± 1.1 ^c^	1.97 ± 0.94 ^d^	0.02 ± 0.62 ^d^	N.D.
Chrysazin	1.16 ± 0.65 ^a,b^	0.62 ± 0.44 ^c^	0.77 ± 0.40 ^c^	0.7 ± 1.4 ^d^	1.75 ± 0.62 ^d^	N.D.
Anthraquinone	0.2 ± 1.9 ^b^	0.5 ± 1.3 ^c^	1.8 ± 2.3 ^c^	16.5 ± 2.1 ^c^	47.2 ± 2.7 ^c^	N.D.

*^a^* Data are expressed as the mean ± SD (*n* = 3). N.D. indicates ‘not determined’ due to lack of a dose response. Values in each column with the same letter are not significantly different between groups at *p* < 0.05.

**Table 4:** Hydrogen peroxide scavenging capacities of BHA and anthraquinones *^a^*.

Sample	H_2_O_2_ Scavenging Activity (%)					
	1 µM	10 µM	50 µM	100 µM	250 µM	IC_50_ (µM)
BHA	9.0 ± 4.6 ^a^	96.6 ± 1.8 ^a^	102.2 ± 1.3 ^a^	103.3 ± 1.4 ^a^	108.2 ± 1.7 ^b^	4.81 ± 0.32 ^b^
Purpurin	10.0 ± 3.3 ^a^	86.8 ± 1.2 ^b^	98.8 ± 4.2 ^a^	106.6 ± 2.9 ^a^	112.0 ± 1.2 ^a^	5.29 ± 0.25 ^b^
Anthrarufin	−1.6 ± 2.2 ^b^	5.32 ± 0.19 ^c^	20.88 ± 0.76 ^b^	28.2 ± 1.2 ^b^	57.2 ± 2.6 ^c^	191.1 ± 6.9 ^a^
Chrysazin	−2.2 ± 3.5 ^b^	−1.3 ± 1.9 ^d^	0.3 ± 1.4 ^c^	3.8 ± 1.6 ^d^	27.12 ± 0.83 ^e^	N.D.
Anthraquinone	0.2 ± 1.9 ^b^	0.5 ± 1.3 ^d^	1.8 ± 2.3 ^c^	16.5 ± 2.1 ^c^	47.2 ± 2.7 ^d^	N.D.

*^a^* Data are expressed as the mean ± SD (*n* = 3). Values in each column with the same letter are not significantly different between groups at *p* < 0.05.

**Table 5:** Reducing powers of BHA and anthraquinones *^a^*.

Sample	Reductive Potential (O.D 700 nm)				
	1 µM	10 µM	50 µM	100 µM	250 µM
BHA	0.019 ± 0.001 ^b^	0.075 ± 0.002 ^b^	0.324 ± 0.004 ^b^	0.605 ± 0.003 ^b^	1.352 ± 0.033 ^b^
Purpurin	0.021 ± 0.000 ^a^	0.082 ± 0.001 ^a^	0.351 ± 0.003 ^a^	0.665 ± 0.002 ^a^	1.452 ± 0.018 ^a^
Anthrarufin	0.012 ± 0.000 ^e^	0.02 ± 0.005 ^c^	0.016 ± 0.000 ^d^	0.035 ± 0.001 ^c^	0.056 ± 0.001 ^c^
Chrysazin	0.015 ± 0.000 ^c^	0.015 ± 0.000 ^d^	0.017 ± 0.000 ^cd^	0.015 ± 0.000 ^e^	0.017 ± 0.012 ^d^
Anthraquinone	0.014 ± 0.000 ^d^	0.014 ± 0.002 ^d^	0.02 ± 0.001 ^c^	0.024 ± 0.004 ^d^	0.027 ± 0.001 ^cd^

*^a^* Data are expressed as the mean ± SD (*n* = 3). Values in each column with the same letter are not significantly different between groups at *p* < 0.05.

**Table 6:** Ferrous ion chelating activities of EDTA, BHA, and anthraquinones *^a^*.

Sample	Metal Ion Chelating Activity (%)					
	1 µM	10 µM	50 µM	100 µM	250 µM	IC_50_ (µM)
EDTA	1.60 ± 0.15 ^a^	1.75 ± 0.13 ^a^	10.46 ± 0.40 ^a^	57.02 ± 0.13 ^a^	99.91 ± 0.05 ^a^	91.46 ± 0.17
BHA	−0.20 ± 0.50 ^b^	−1.46 ± 0.83 ^c^	−1.58 ± 0.25 ^e^	−2.41 ± 0.30 ^f^	−1.12 ± 0.69 ^f^	N.D.
Purpurin	−0.83 ± 0.41 ^bc^	2.01 ± 0.45 ^a^	8.17 ± 0.53 ^b^	12.26 ± 0.91 ^b^	24.2 ± 1.5 ^b^	N.D.
Anthrarufin	−0.20 ± 0.57 ^b^	0.03 ± 0.39 ^b^	3.81 ± 0.54 ^c^	6.9 ± 1.2 ^c^	12.75 ± 0.73 ^d^	N.D.
Chrysazin	−0.92 ± 0.65 ^bc^	−0.80 ± 0.89 ^bc^	2.41 ± 0.79 ^d^	3.95 ± 0.39 ^d^	8.48 ± 0.26 ^e^	N.D.
Anthraquinone	−1.55 ± 0.17 ^c^	−0.11 ± 0.09 ^b^	3.47 ± 0.28 ^c^	0.8 ± 1.0 ^e^	15.30 ± 0.89 ^c^	N.D.

*^a^* Data are expressed as the mean ± SD (*n* = 3). Values in each column with the same letter are not significantly different between groups at *p* < 0.05.
